# Effect of fluid resuscitation on mortality and organ function in experimental sepsis models

**DOI:** 10.1186/cc8179

**Published:** 2009-11-23

**Authors:** Sebastian Brandt, Tomas Regueira, Hendrik Bracht, Francesca Porta, Siamak Djafarzadeh, Jukka Takala, José Gorrasi, Erika Borotto, Vladimir Krejci, Luzius B Hiltebrand, Lukas E Bruegger, Guido Beldi, Ludwig Wilkens, Philipp M Lepper, Ulf Kessler, Stephan M Jakob

**Affiliations:** 1 Department of Anaesthesia and Pain Therapy, Inselspital, Bern University Hospital and University of Bern, CH-3010 Bern, Switzerland; 2 Department of Intensive Care Medicine, Inselspital, Bern University Hospital and University of Bern, CH-3010 Bern, Switzerland; 3 Department of Visceral and Transplant Surgery, Inselspital, Bern University Hospital and University of Bern, CH-3010 Bern, Switzerland; 4 Department of Pediatric Surgery, Inselspital, Bern University Hospital and University of Bern, CH-3010 Bern, Switzerl; 5 Institute of Pathology, University of Bern, Murtenstrasse 31, CH-3010 Bern, Switzerland

## Abstract

**Introduction:**

Several recent studies have shown that a positive fluid balance in critical illness is associated with worse outcome. We tested the effects of moderate vs. high-volume resuscitation strategies on mortality, systemic and regional blood flows, mitochondrial respiration, and organ function in two experimental sepsis models.

**Methods:**

48 pigs were randomized to continuous endotoxin infusion, fecal peritonitis, and a control group (n = 16 each), and each group further to two different basal rates of volume supply for 24 hours [moderate-volume (10 ml/kg/h, Ringer's lactate, n = 8); high-volume (15 + 5 ml/kg/h, Ringer's lactate and hydroxyethyl starch (HES), n = 8)], both supplemented by additional volume boli, as guided by urinary output, filling pressures, and responses in stroke volume. Systemic and regional hemodynamics were measured and tissue specimens taken for mitochondrial function assessment and histological analysis.

**Results:**

Mortality in high-volume groups was 87% (peritonitis), 75% (endotoxemia), and 13% (controls). In moderate-volume groups mortality was 50% (peritonitis), 13% (endotoxemia) and 0% (controls). Both septic groups became hyperdynamic. While neither sepsis nor volume resuscitation strategy was associated with altered hepatic or muscle mitochondrial complex I- and II-dependent respiration, non-survivors had lower hepatic complex II-dependent respiratory control ratios (2.6 +/- 0.7, vs. 3.3 +/- 0.9 in survivors; P = 0.01). Histology revealed moderate damage in all organs, colloid plaques in lung tissue of high-volume groups, and severe kidney damage in endotoxin high-volume animals.

**Conclusions:**

High-volume resuscitation including HES in experimental peritonitis and endotoxemia increased mortality despite better initial hemodynamic stability. This suggests that the strategy of early fluid management influences outcome in sepsis. The high mortality was not associated with reduced mitochondrial complex I- or II-dependent muscle and hepatic respiration.

## Introduction

Severe sepsis and septic shock are major causes of death in intensive care patients [1,2]. Most deaths from septic shock can be attributed to either cardiovascular or multiorgan failure [3]. The causes of organ dysfunction and failure are unclear, but inadequate tissue perfusion, systemic inflammation, and direct metabolic changes at the cellular level are all likely to contribute [4-6].

Fluid resuscitation is a major component of cardiovascular support in early sepsis. Although the need for fluid resuscitation in sepsis is well established [7], the goals and components of this treatment are still a matter of debate. Several recent studies have shown that a positive fluid balance in critical illness is strongly associated with a higher severity of organ dysfunction and with worse outcome [8-14]. It is unclear whether this is the primary consequence of fluid therapy *per se*, or reflects the severity of illness.

We hypothesized that the fluid resuscitation strategy has an impact on sepsis-related metabolic and cellular alterations, and outcome in sepsis. To test this hypothesis, we used two different basal rates of volume supply (to mimic 'restrictive' and 'wet' approaches), supplemented by additional volume boli, when clinically relevant and commonly used physiological variables such as urinary output or filling pressures decreased. We measured the effects of these two volume approaches on systemic and regional blood flows, organ function and mortality. As no experimental model can directly be extrapolated to clinical sepsis and the effects of fluid resuscitation may be model-dependent [15,16], two different sepsis models - fecal peritonitis and endotoxemia - were studied.

## Materials and methods

The study was performed in accordance with the National Institutes of Health guidelines for the care and use of experimental animals and with the approval of the Animal Care Committee of the Canton of Bern, Switzerland.

The experimental design included two factors: the model of sepsis (control, peritonitis, endotoxemia) and the strategy of fluid resuscitation (moderate volume or high volume). A full factorial design with six experimental groups was used.

### Animal preparation and experimental setting

Pigs of both sexes (weight: median 41 kg; range 38 to 44 kg) were fasted overnight. They were then premedicated, anesthetized with pentobarbital, intubated endotracheally and ventilated (volume control mode; Servo ventilator 900 C; Siemens-Elema^®^, Solna, Sweden) with 5 cm H_2_O positive end-expiratory pressure. Anesthesia was maintained with pentobarbital (7 mg/kg/h) and fentanyl (25 μg/kg/h during operation and 3 μg/kg/h afterwards), and pancuronium (1 mg/kg/h) was used for muscle relaxation. A single dose of 1.5 g cefuroxime was injected before surgery. An esophageal Doppler probe (Deltex^®^, Chichester, UK) was inserted, and catheters for pressure measurement and blood sampling were placed into the carotid, hepatic and pulmonary arteries, and into the jugular, hepatic, portal, renal and mesenteric veins. Ultrasound Doppler flow probes (Transonic^® ^System Inc., Ithaca, NY, USA) were positioned around the carotid, superior mesenteric, splenic and hepatic arteries, and celiac trunk and portal vein. Laser Doppler needle and surface probes (Optronics^®^, Oxford, UK) were inserted into the liver and kidney, and fixed on the surface of gastric and jejunal mucosa and the kidney. More details on the surgical procedure are described in the supplement [see Additional Data File 1].

### Experimental protocol

After surgery, approximately 12 hours was allowed for hemodynamic stabilization. During this period, Ringer's lactate at 10 ml/kg/h was infused to keep hemodynamic stability. The animals were then randomized into six groups (eight pigs in each): control, fecal peritonitis, or endotoxin, each with either high (15 ml/kg/hr Ringer's lactate and 5 ml/kg/hr hydroxyethyl starch (HES) 130/04, 6% (Voluven^®^, Fresenius, Stans, Switzerland)) or moderate volume fluid resuscitation (10 mL/kg/hr Ringer's lactate).

In the peritonitis groups, 1 g per kg of autologous feces, dissolved in warmed glucose solution, was instilled in the abdominal cavity. In the other groups, the same amount of sterile glucose solution was instilled. The intraperitoneal drains were clamped during the first six hours. In the endotoxin groups, endotoxin (lipopolysaccharide from *Escherichia coli *0111:B4, 20 mg/l in 5% dextrose; Sigma^®^, Steinheim, Germany) was infused into the right atrium. The effect of endotoxin was judged by the magnitude of pulmonary artery pressure. Initially, endotoxin was infused at 0.4 μg/kg/h until mean pulmonary arterial pressure reached 35 mmHg and the animals became hypotensive. The endotoxin infusion was then stopped, and if arterial hypotension persisted (mean arterial pressure below 60 mmHg), 50 ml of HES was administered. If an arterial blood pressure of more than 55 mmHg could not be restored, boluses of adrenaline (5 to 10 μg/bolus) were injected to prevent acute right heart failure and death. Adrenaline was only used to treat hypotension within one hour of the onset of pulmonary artery hypertension. If mean pulmonary pressure subsequently decreased below 30 mmHg, the endotoxin infusion was restarted (0.1 μg/kg/h) and increased hourly by 30%, if necessary, to maintain mean pulmonary artery pressure at 25 to 30 mmHg. After eight hours of endotoxin infusion, the infusion rate was kept constant.

Throughout the experiment (including the postoperative stabilization period), the volume status was evaluated clinically every hour, and if signs of hypovolemia became evident (pulmonary artery occlusion pressure ≤ 5 mmHg or urinary output ≤ 0.5 mL/kg/hour), additional 50 ml boluses of HES were given regardless of study group. Fluid boluses were repeated under stroke volume monitoring with esophageal Doppler for as long as the stroke volume was increased by 10% or more. For the validity of esophageal Doppler with respect to cardiac output measurement by thermodilution see Dark and Singer [17]. To maintain the differences between high- and moderate-volume groups, maximal additional volume was restricted to 100 ml per hour in all groups. Vasopressors were not used. If necessary, 50% glucose solution was administered to maintain blood glucose of 3.5 to 6 mmol/l, and the standard infusion rate was adjusted to maintain unchanged basal volume supply.

The quadriceps muscle was biopsied at baseline, after six hours, and at the end of the experiment, and the liver was biopsied at the end of the experiment, for mitochondrial function measurement [see Additional Data File 1].

The animals were followed until 24 hours after randomization or until death, if earlier. After 24 hours, the animals were euthanized with an overdose of potassium chloride. Blood sampling, histological analysis and interpretation of causes of mortality are described in the online supplement [see Additional Data File 1].

### Statistical analysis

The SPSS 13.0 software package (SPSS Inc.^®^, Chicago, IL, USA) was used for statistical analysis. Normal distribution was assessed by the Kolmogorov-Smirnov test.

Survival proportions between the groups were analyzed with the log rank test, followed by *post-hoc *log-rank tests for groups 'low volume' vs. 'high volume' and for groups 'endotoxemia' vs. 'fecal peritonitis' vs. 'controls'. Differences between groups were assessed by multivariate analysis of variance for repeated measures using one dependent variable, two between-subject factors -- model (control, endotoxemia, peritonitis) and volume (moderate, high) -- and one within-subject factor (time). Significant time-volume and time-model interactions were considered as effects of volume resuscitation and experimental model, respectively. If significant interactions occurred, analysis of variance (ANOVA) for repeated measures was performed in the individual involved groups to assess where changes occurred.

Fluid input and balance were compared with one-way ANOVA. The Tukey *post-hoc *test was performed to assess differences between the models. For hepatic mitochondrial analysis, univariate analysis of variance was used. Significant effects of the fixed factors *model *and *volume *were further analyzed *post hoc *with the independent t-test. For comparison of mitochondrial function between survivors and non-survivors, an analysis of variance for repeated measures was used for muscle mitochondria and an independent t-test for liver mitochondria. Statistical significance was considered at *P *< 0.05. In *post-hoc *testing, the difference between groups with the lowest *P *value (even when >0.05) was considered responsible for the observed significant results in primary testing. Data are expressed as mean ± standard deviation.

## Results

### Fluid balance

**Figure 1:**
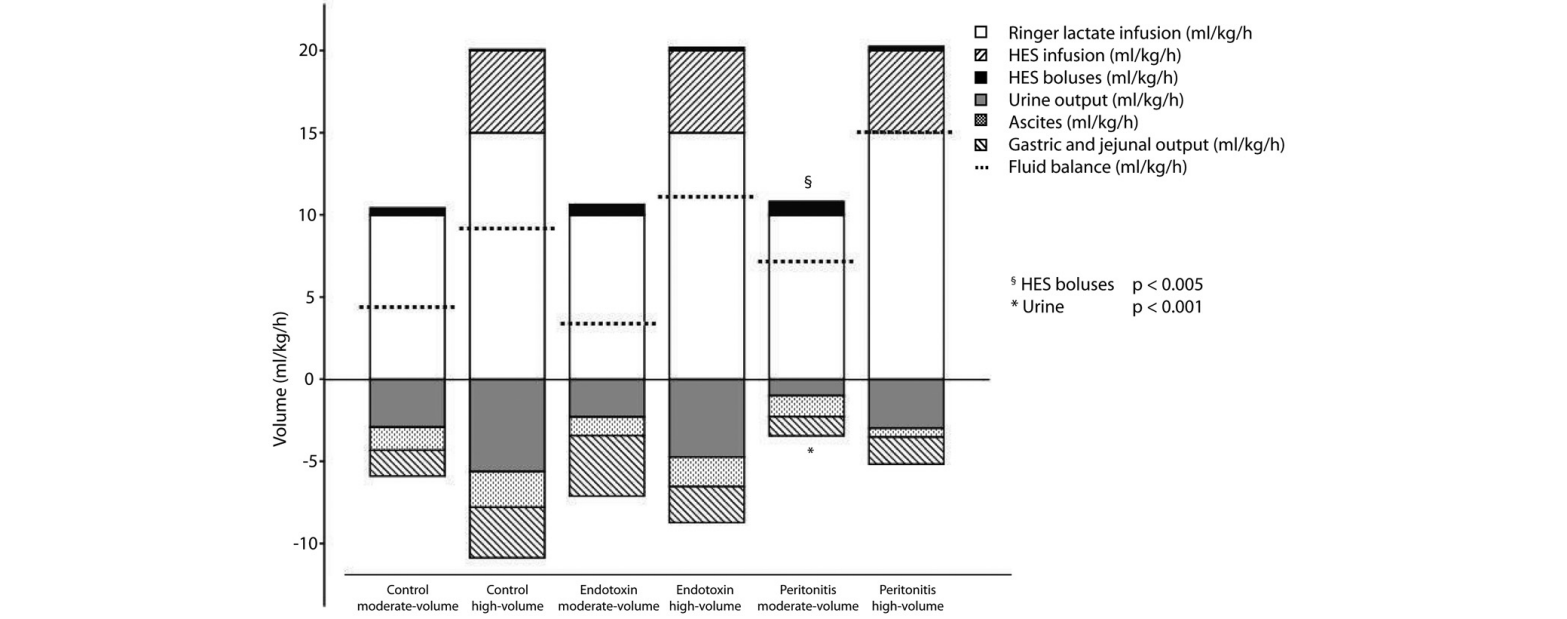
Continuous and bolus inputs and urine, gastric and ascites outputs for each group. Total fluid administration; balance: high-volume groups vs. moderate volume groups *P *= 0.001 (one-way analysis of variance). Diuresis (*) and additional hydroxyethyl starch (HES) boluses (§: peritonitis moderate-volume *P *< 0.001 (Tukey).

The three moderate-volume groups received an average of 11.0, and the high-volume groups 2.4 boli of additional volume. The total fluid balance was markedly higher in the high-volume groups (*P *< 0.001; Figure 1). Both peritonitis groups exhibited significantly higher fluid balances than their matching other groups (*P *= 0.001).

### Mortality

**Figure 2:**
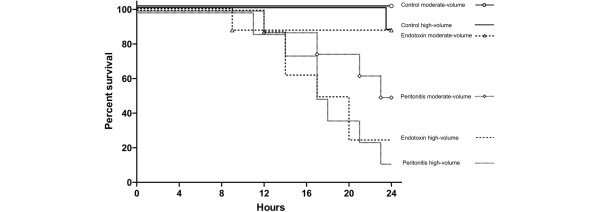
Survival curves of all experimental groups. log rank test: *P *< 0.001. The cause of death is also shown for each pig.

Eight animals had to be excluded from the analysis due to acute right-heart failure and death within minutes after the start of endotoxin infusion (n = 7) and gut perforation with rapid development of septic shock (n = 1). We found differences in mortality (*P *< 0.001), with highest values in the peritonitis high-volume (n = 7; 88%) and endotoxin high-volume (n = 6, 75%) groups. Mortality was higher in high- vs. low-volume groups, and in septic vs. control groups (*P *< 0.01, both), but did not differ between endotoxemia and fecal peritonitis groups. The respective median survival times were 17.5 and 16 hours. Mortality was 50% (n = 4) in the peritonitis moderate-volume group and 12.5% (n = 1) in the endotoxin moderate-volume group, with median survival times of 23.5 and 24 hours, respectively. One animal in the control high-volume group died at 23.5 hours, while all moderate-volume control pigs survived until the end of the experiment (Figure 2).

### Systemic hemodynamics, oxygen transport and lactate concentrations

Both the experimental model and volume management modified the hemodynamic response, that is, cardiac output, heart rate, systemic and pulmonary artery pressures, and filling pressures (Tables 1 and 2). The peritonitis groups became hypotensive (*P *< 0.002) and the endotoxin groups transiently hypertensive (*P *= 0.001). Cardiac output increased in both septic groups (endotoxin: *P *= 0.002; peritonitis: *P *= 0.04; Table 1). Mean pulmonary artery and pulmonary artery occlusion pressures increased in all groups (both *P *< 0.001). At the end of the experiment, pulmonary artery pressures were highest in both septic high-volume groups (*P *= 0.001), and pulmonary artery occlusion pressures were highest in the peritonitis high-volume group (*P *= 0.008). Mixed venous saturation decreased in both peritonitis groups (*P *= 0.008; Table 2). Arterial lactate concentration increased in endotoxin (*P *= 0.04) and in peritonitis pigs (*P *= 0.001; Table 2). Oxygen transport data are indicated in the electronic supplement [see Table S1 in Additional Data File 2].

**Table 1:** Systemic hemodynamics

Variable	Group	N	Intra-operative	Baseline	3 hours	6 hours	12 hours	End	Interactions	*P*
Cardiac index (ml/kg/min)									Time × model effect:	*0.02*
	C 10 ml/kg	8	n. a.	89 ± 14	88 ± 21	93 ± 22	100 ± 32	103 ± 24		
	C 20 ml/kg	8	n. a.	73 ± 24	88 ± 10	92 ± 11	96 ± 22	99 ± 14		
	E 10 ml/kg	7	n. a.	75 ± 17	69 ± 21	84 ± 25	98 ± 29	113 ± 32		
	E 20 ml/kg	8	n. a.	87 ± 19	83 ± 24	106 ± 33	130 ± 37	117 ± 38	ANOVArm E:	*0.002*
	P 10 ml/kg	8	n. a.	86 ± 17	92 ± 28	105 ± 26	87 ± 26	94 ± 13		
	P 20 ml/kg	8	n. a.	82 ± 12	113 ± 31	103 ± 21	108 ± 24	133 ± 73	ANOVArm P:	*0.04*

Heart rate (beats/min)									Time × model effect:	*0.001*
	C 10 ml/kg	8	116 ± 19	114 ± 38*	129 ± 40	138 ± 45	147 ± 42	138 ± 27	ANOVArm C:	*0.04*
	C 20 ml/kg	8	126 ± 24	112 ± 25*	107 ± 18	124 ± 33	125 ± 29	135 ± 37		
	E 10 ml/kg	7	119 ± 20	99 ± 12*	114 ± 28	130 ± 28	153 ± 27	166 ± 20	ANOVArm E:	*0.002*
	E 20 ml/kg	8	122 ± 16	111 ± 22*	99 ± 15	117 ± 25	137 ± 36	136 ± 33		
	P 10 ml/kg	8	115 ± 19	114 ± 12*	164 ± 24	186 ± 27	165 ± 37	148 ± 36	ANOVArm P:	*0.001*
	P 20 ml/kg	8	117 ± 13	99 ± 11*	158 ± 37	175 ± 20	154 ± 35	156 ± 47		

Stroke volume index (ml/kg/beat)									Time × volume effect:	*0.03*
	C 10 ml/kg	8	n. a.	0.8 ± 0.2	0.7 ± 0.3	0.7 ± 0.3	0.7 ± 0.2	0.8 ± 0.3	ANOVArm moderate-volume:	*0.018*
	C 20 ml/kg	8	n. a.	0.7 ± 0.3	0.8 ± 0.1	0.8 ± 0.2	0.8 ± 0.2	0.8 ± 0.2		
	E 10 ml/kg	7	n. a.	0.8 ± 0.1	0.6 ± 0.2	0.7 ± 0.2	0.7 ± 0.3	0.7 ± 0.2		
	E 20 ml/kg	8	n. a.	0.8 ± 0.2	0.9 ± 0.3	0.9 ± 0.3	1.0 ± 0.4	1.0 ± 0.5		
	P 10 ml/kg	8	n. a.	0.8 ± 0.1	0.6 ± 0.2	0.6 ± 0.1	0.6 ± 0.3	0.7 ± 0.2		
	P 20 ml/kg	8	n. a.	0.8 ± 0.1	0.8 ± 0.3	0.6 ± 0.2	0.7 ± 0.2	0.9 ± 0.5		

Mean arterial pressure (mmHg)									Time × model effect: Time × volume effect:	*0.001* *0.03*
	C 10 ml/kg	8	91 ± 13	71 ± 7^#^	69 ± 14	72 ± 12	75 ± 5	72 ± 14		
	C 20 ml/kg	8	92 ± 5	69 ± 11^#^	75 ± 15	77 ± 15	83 ± 15	76 ± 24	ANOVArm high-volume:	*0.001*
	E 10 ml/kg	7	97 ± 8	69 ± 8^#^	86 ± 12	76 ± 14	78 ± 11	80 ± 11		
	E 20 ml/kg	8	99 ± 19	70 ± 13^#^	105 ± 8	102 ± 16	86 ± 18	74 ± 23	ANOVArm E:	*0.001*
	P 10 ml/kg	8	87 ± 13	69 ± 10^#^	75 ± 14	64 ± 10	66 ± 15	49 ± 20		
	P 20 ml/kg	8	86 ± 16	74 ± 26^#^	86 ± 23	83 ± 23	76 ± 27	61 ± 25	ANOVArm P:	*0.002*

Mean pulmonary artery pressure (mmHg)									Time × model effect: Time × volume effect:	*0.003* *0.01*
	C 10 ml/kg	8	n. a.	17 ± 4	19 ± 6	18 ± 3	20 ± 4	25 ± 3	ANOVArm moderate-volume:	*0.001*
	C 20 ml/kg	8	n. a.	18 ± 4	20 ± 5	19 ± 5	23 ± 7	29 ± 6	ANOVArm C:	*0.001*
	E 10 ml/kg	7	n. a.	17 ± 2	27 ± 7	25 ± 6	22 ± 5	26 ± 5	ANOVArm E:	*0.001*
	E 20 ml/kg	7	n. a.	17 ± 3	33 ± 12	27 ± 6	29 ± 13	34 ± 11		
	P 10 ml/kg	8	n. a.	16 ± 3	23 ± 6	20 ± 3	21 ± 4	24 ± 3	ANOVArm P:	*0.001*
	P 20 ml/kg	7	n. a.	18 ± 3	25 ± 5	25 ± 4	29 ± 6	36 ± 6	ANOVArm high-volume:	*0.001*

Values are mean ± standard deviation. C = controls; E = endotoxin; P = peritonitis

early intraoperative vs. baseline * *P *< 0.0001, ^# ^*P *< 0.007

**Table 2:** Filling pressures, mixed venous oxygen saturation and arterial lactate concentrations

Variable	Group	N	Baseline	3 hours	6 hours	12 hours	End	Interactions	*P*
Central venous pressure (mmHg)									
	C 10 ml/kg	8	4 ± 2	4 ± 2	5 ± 2	5 ± 1	7 ± 2	ANOVArm moderate-volume:	*0.001*
	C 20 ml/kg	8	4 ± 2	6 ± 2	5 ± 2	7 ± 4	10 ± 5	ANOVArm C:	*0.001*
	E 10 ml/kg	7	4 ± 2	4 ± 2	5 ± 2	5 ± 2	6 ± 3	ANOVArm E:	*0.001*
	E 20 ml/kg	8	3 ± 2	6 ± 3	7 ± 3	7 ± 2	9 ± 1		
	P 10 ml/kg	8	3 ± 2	3 ± 1	4 ± 1	6 ± 2	7 ± 2	ANOVArm P:	*0.001*
	P 20 ml/kg	8	5 ± 3	6 ± 3	8 ± 4	10 ± 3	14 ± 3	ANOVArm high-volume:	*0.001*

Pulmonary artery occlusion pressure (mmHg)									
	C 10 ml/kg	8	4 ± 2	5 ± 2	5 ± 2	5 ± 2	8 ± 2	ANOVArm:	*0.001*
	C 20 ml/kg	8	5 ± 3	6 ± 3	6 ± 2	7 ± 4	10 ± 5	ANOVArm:	*0.013*
	E 10 ml/kg	7	5 ± 1	5 ± 2	5 ± 2	5 ± 2	7 ± 4	ANOVArm:	*0.10*
	E 20 ml/kg	7	5 ± 3	9 ± 5	7 ± 4	8 ± 5	10 ± 3	ANOVArm:	*0.018*
	P 10 ml/kg	8	4 ± 1	4 ± 1	5 ± 2	6 ± 2	8 ± 2	ANOVArm:	*0.001*
	P 20 ml/kg	7	7 ± 2	7 ± 2	8 ± 3	10 ± 2	17 ± 8	ANOVArm:	*0.008*

Mixed venous saturation (%)								Time × model effect:	*0.009*
	C 10 ml/kg	8	55 ± 6	54 ± 11	55 ± 1	55 ± 8	57 ± 7		
	C 20 ml/kg	8	49 ± 7	59 ± 5	59 ± 4	60 ± 1	55 ± 18		
	E 10 ml/kg	7	49 ± 6	51 ± 5	55 ± 6	57 ± 3	55 ± 8	ANOVArm E:	*0.013*
	E 20 ml/kg	7	49 ± 5	49 ± 11	60 ± 8	66 ± 2	56 ± 11		
	P 10 ml/kg	8	53 ± 7	59 ± 4	58 ± 7	55 ± 6	47 ± 13	ANOVArm P:	*0.008*
	P 20 ml/kg	7	46 ± 1	57 ± 12	56 ± 14	57 ± 9	43 ± 24		

Arterial lactate (mmol/l)								Time × model effect:	*0.046*
	C 10 ml/kg	8	0.6 ± 0.2	0.5 ± 0.2	0.7 ± 0.5	0.6 ± 0.1	0.7 ± 0.2		
	C 20 ml/kg	8	0.6 ± 0.1	0.6 ± 0.2	0.6 ± 0.1	0.6 ± 0.1	1.0 ± 1.0		
	E 10 ml/kg	7	0.7 ± 0.1	1.2 ± 0.7	0.9 ± 0.4	0.8 ± 0.3	0.9 ± 0.5	ANOVArm E:	*0.04*
	E 20 ml/kg	8	0.7 ± 0.1	1.0 ± 0.3	0.9 ± 0.3	1.0 ± 0.2	1.0 ± 0.3		
	P 10 ml/kg	8	0.6 ± 0.2	1.4 ± 0.6	1.5 ± 0.6	1.1 ± 0.4	1.5 ± 0.6	ANOVArm P:	*0.001*
	P 20 ml/kg	8	0.8 ± 0.6	1.1 ± 0.6	1.1 ± 0.6	1.1 ± 0.3	1.4 ± 0.6		

Values are mean ± standard deviation. C = controls; E = endotoxin; P = peritonitis

### Mitochondrial function

Sepsis had only limited effects on hepatic mitochondrial respiration [see Table S2 in Additional Data File 2 and Figure S1 in Additional Data File 3]. Complex I-dependent resting respiration (state 4) was lower in endotoxin animals in comparison with controls [see Figure S1 in Additional Data File 3], and the complex I-dependent maximal ATP production was lower in peritonitis moderate vs. high volume [see Table S2 in Additional Data File 2]. Hepatic vein lactate/pyruvate ratios were not different between the groups [see Figure S2 in Additional Data File 3].

*Skeletal muscle *mitochondrial respiration was not affected by sepsis [see Table S3 in Additional Data File 2 and Figure S3 in Additional Data File 3]. Complex I-dependent maximal mitochondrial oxygen consumption (state 3) was higher in high-volume animals at six hours [see Figure S3 in Additional Data File 3]. Muscle ATP content decreased in septic moderate-volume animals [see Table S3 in Additional Data File 2]. Muscle ATP/ADP ratio was lower in peritonitis moderate vs. high-volume groups [see Table S3 in Additional Data File 2].

### Lungs

The oxygenation index (partial pressure of arterial oxygen to fraction of inspired oxygen) decreased in all groups over the course of the experiment, but most in the peritonitis groups (*P *= 0.001; Table 3). The respiratory plateau pressure increased in all groups, with the highest values in control and peritonitis high-volume animals (*P *= 0.04; Table 3). The dynamic compliance of the respiratory system decreased in all groups, without differences related to volume or model. Lung histology revealed the presence of colloid plaques and atelectases in all groups of animals [see Figures S4 and S5 in Additional Data File 3]. Colloid plaques tended to be more frequently present in the high-volume groups (84%) in comparison with their respective moderate-volume groups (59%). Atelectases were present in 50% or more of the animals of all groups.

**Table 3:** Respiratory parameters

Variable	Group	N	Baseline	3 hours	6 hours	12 hours	End	Interactions	*P*
Dynamic compliance								Time effect:	*0.001*
	C 10 ml/kg	8	28 ± 6	25 ± 8	26 ± 7	25 ± 8	17 ± 5		
	C 20 ml/kg	8	30 ± 7	25 ± 8	26 ± 6	21 ± 5	14 ± 5		
	E 10 ml/kg	7	31 ± 7	27 ± 5	28 ± 6	24 ± 5	18 ± 4		
	E 20 ml/kg	8	32 ± 3	25 ± 3	24 ± 3	22 ± 5	22 ± 5		
	P 10 ml/kg	8	28 ± 2	24 ± 6	20 ± 3	20 ± 2	15 ± 2		
	P 20 ml/kg	8	32 ± 8	25 ± 6	21 ± 3	18 ± 2	14 ± 6		

Plateau pressure (cmH_2_O)								Time × volume effect:	*0.043*
	C 10 ml/kg	8	18 ± 2	19 ± 2	20 ± 3	19 ± 4	24 ± 4		
	C 20 ml/kg	8	18 ± 2	20 ± 2	20 ± 2	22 ± 4	28 ± 8		
	E 10 ml/kg	7	16 ± 3	18 ± 4	18 ± 4	17 ± 4	22 ± 6	ANOVArm moderate-volume:	*0.001*
	E 20 ml/kg	7	15 ± 5	19 ± 7	21 ± 6	18 ± 6	21 ± 7		
	P 10 ml/kg	8	17 ± 3	19 ± 3	20 ± 4	22 ± 2	24 ± 5		
	P 20 ml/kg	7	16 ± 4	19 ± 6	22 ± 5	22 ± 6	28 ± 6	ANOVArm high-volume:	*0.001*

Oxygenation index (mmHg/%)								Time × model effect:	*0.026*
	C 10 ml/kg	8	434 ± 67	394 ± 92	384 ± 97	346 ± 67	212 ± 97	ANOVArm C:	*0.001*
	C 20 ml/kg	8	456 ± 48	412 ± 80	424 ± 48	347 ± 106	236 ± 122		
	E 10 ml/kg	7	477 ± 33	418 ± 44	401 ± 57	352 ± 101	208 ± 116	ANOVArm E:	*0.001*
	E 20 ml/kg	7	447 ± 44	313 ± 88	291 ± 102	252 ± 110	170 ± 139		
	P 10 ml/kg	8	449 ± 29	356 ± 54	300 ± 69	317 ± 99	217 ± 106	ANOVArm P:	*0.001*
	P 20 ml/kg	8	412 ± 61	292 ± 104	247 ± 74	193 ± 112	63 ± 12		

Values are mean ± standard deviation. C = controls; E = endotoxin; P = peritonitis

### Kidney

Renal artery blood flow decreased in both peritonitis groups (*P *= 0.024) [see Table S4 in Additional Data File 2]. Urinary output was highest in control high-volume and endotoxin high-volume groups (Figure 1). In contrast, peritonitis high-volume pigs produced less urine, comparable to control moderate-volume pigs. The lowest diuresis was observed in peritonitis moderate-volume pigs (Figure 1; *P *< 0.001). Base excess decreased in both peritonitis groups but not in the other groups (*P *= 0.001) [see Table S1 in Additional Data File 2], while serum creatinine decreased in controls (*P *= 0.007) and high-volume groups (*P *= 0.04; Table 4).

**Table 4:** Laboratory parameters

Variable	Group	N	Baseline	End	Interactions	*P*
Creatinine kinase - MB (U/L)					Time × volume effect:	*0.006*
	Control 10 ml/kg	8	0.9 ± 0.1	1.0 ± 0.2		
	Control 20 ml/kg	8	0.9 ± 0.1	1.2 ± 0.2	ANOVArm high-volume:	*0.001*
	Endotoxin 10 ml/kg	8	1.0 ± 0.2	1.0 ± 0.2		
	Endotoxin 20 ml/kg	7	1.0 ± 0.2	1.1 ± 0.3		
	Peritonitis 10 ml/kg	8	1.1 ± 0.2	1.1 ± 0.3		
	Peritonitis 20 ml/kg	8	0.8 ± 0.3	1.3 ± 0.2		

Creatinine (μmol/L)					Time × model effect: Time × volume effect:	*0.014* *0.029*
	Control 10 ml/kg	8	87 ± 18	78 ± 17	ANOVArm Control:	*0.007*
	Control 20 ml/kg	8	99 ± 13	74 ± 24	ANOVArm high-volume:	*0.04*
	Endotoxin 10 ml/kg	8	86 ± 21	79 ± 16		
	Endotoxin 20 ml/kg	7	85 ± 15	74 ± 10		
	Peritonitis 10 ml/kg	8	81 ± 10	114 ± 31		
	Peritonitis 20 ml/kg	8	82 ± 17	76 ± 36		

ALAT (U/L)					Time × volume effect:	*0.001*
	Control 10 ml/kg	8	18.1 ± 4.3	14.8 ± 4.5		
	Control 20 ml/kg	8	20.5 ± 10.5	11.3 ± 10.6	ANOVArm high-volume:	*0.001*
	Endotoxin 10 ml/kg	8	17 ± 5.2	15.1 ± 4.3		
	Endotoxin 20 ml/kg	7	19 ± 5.7	11.4 ± 2.4		
	Peritonitis 10 ml/kg	8	16.9 ± 6.4	16.7 ± 11.2		
	Peritonitis 20 ml/kg	8	19.5 ± 9.2	11.3 ± 5		

ASAT (U/L)						
	Control 10 ml/kg	8	84 ± 31	60 ± 27		
	Control 20 ml/kg	8	114 ± 57	76 ± 23		
	Endotoxin 10 ml/kg	8	96 ± 23	86 ± 44		
	Endotoxin 20 ml/kg	7	136 ± 84	117 ± 23		
	Peritonitis 10 ml/kg	8	104 ± 43	129 ± 88		
	Peritonitis 20 ml/kg	8	101 ± 50	100 ± 55		

Values are mean ± standard deviation. ALAT = alanine aminotransferase; ASAT = aspartate aminotransferase.

**Figure 3:**
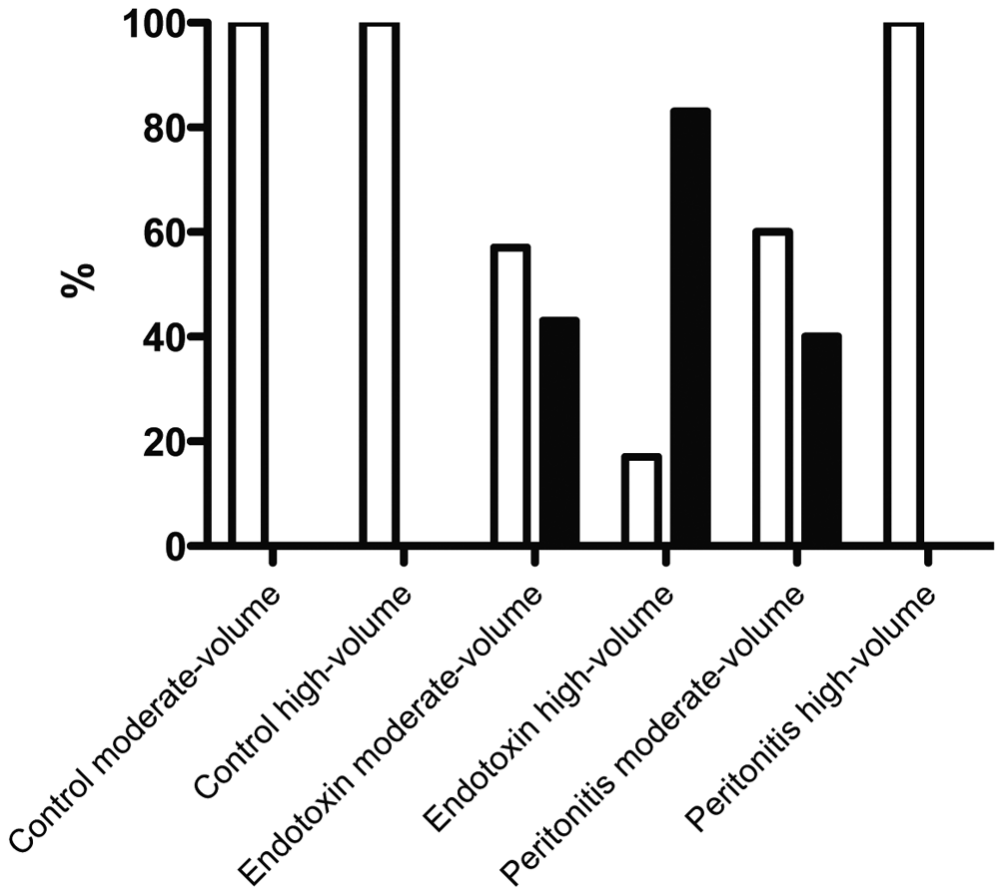
Histogram showing kidney histology and severity of damage

**Figure 4:**
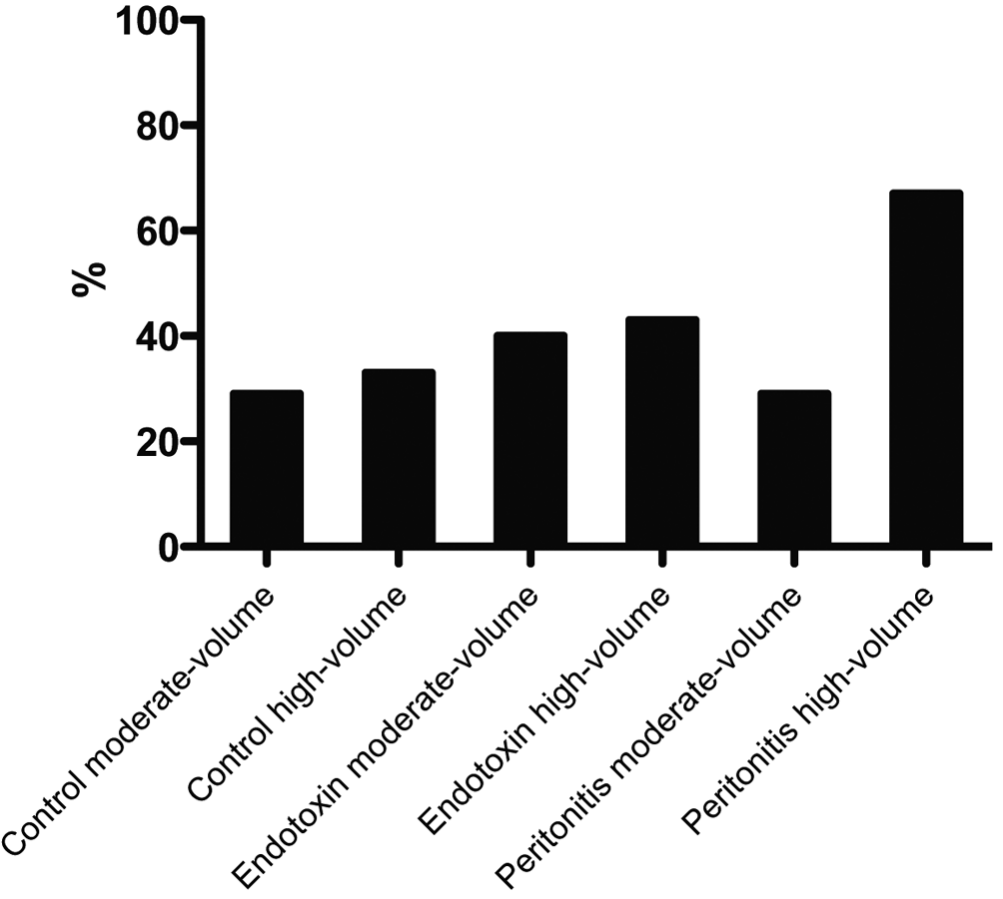
Histogram showing kidney histology and distribution of colloid plaques

Histology revealed severe damage in five of six endotoxin high-volume animals (83%) and in 30% to 40% of the animals in the endotoxin and peritonitis moderate-volume groups (Figure 3). Storage of starch (HES) in the tissues was detectable as a purple fluid in H&E-stained tissue sections, as confirmed by positive Periodic acid-Schiff staining. This fluid was mainly found in dilated tubules. There was no predilection for one of the groups (Figure 4).

### Liver

Hepatic artery blood flow was mainly influenced by the model, with flows increasing to highest levels in the endotoxin groups (*P *= 0.006) [see Table S4 in Additional Data File 2]. Serum alanine aminotransferase decreased in all high-volume groups and stayed stable in moderate-volume groups (*P *= 0.001; Table 4). Histology revealed accentuated sinusoidal structures, both local and diffuse vacuolization, and pericentral necrosis [see Figure S6 in Additional Data File 3]. Generalized sinusoidal dilatation was seen only in endotoxin animals, while other histological abnormalities were present in all groups (including controls) in various degrees, showing a tendency to model-specific histological patterns.

### Heart

The serum levels of creatine kinase isoenzyme increased in all high-volume groups and stayed stable in moderate-volume pigs (time × volume *P *= 0.006; Table 4).

## Discussion

The main finding of this study was that high-volume fluid resuscitation including HES increased mortality in sepsis. The increased mortality was observed in both models of fecal peritonitis and endotoxemia. Both these established large-animal sepsis models share many of the features of clinical sepsis, including hypovolemia if untreated, normo- or hyperdynamic circulation with volume resuscitation, high mortality, and signs of progressive organ dysfunction despite cardiovascular and respiratory support.

Despite major differences in volume supply, differences in hemodynamic responses between the groups were either modest or appeared late: the most prominent difference was progressive pulmonary artery hypertension and increased cardiac filling pressures in the high-volume groups, especially in peritonitis. We did not perform echocardiography, so direct evaluation of myocardial function was not possible. In particular the severity of right ventricular dysfunction may have been underestimated. The increased cardiac enzymes in all high-volume groups support the concept that relevant myocardial damage occurred. Fluid loading in septic animals has been shown to induce a large reduction in vascular tone, which could be attenuated by inhibition of nitric oxide synthesis [18]. It is conceivable to argue that high amounts of volume can promote vascular leak and interstitial edema in septic states by releasing nitric oxide and/or other vasodilating agents. This effect would be even more exaggerated when filling pressures increase as an effect of cardiac dysfunction. In our study, lung dysfunction, reflected in impaired oxygenation index and mechanics, was the cause of approximately every third death in the high-volume septic groups and none in the moderate-volume groups. Renal perfusion was also predominantly affected in the high-volume septic animals; especially in peritonitis, despite high cardiac output and relatively well-preserved mean arterial pressure.

The criteria for and targets of fluid management in sepsis are controversial. In clinical sepsis, recent guidelines - based mainly on expert opinions (Surviving Sepsis Campaign) - have recommended fluid administration to restore cardiac filling pressures to at least 12 mmHg during mechanical ventilation [19]. In mechanically ventilated patients or patients with known pre-existing decreased ventricular compliance, central venous pressure targets of 12 to 15 mmHg have been suggested [20]. In clinical sepsis trials where fluid was administered to optimize hemodynamics, central venous pressures of up to 22 mmHg have been reached [21]. In the present study, only the high-volume groups reached levels recommended by the Surviving Sepsis campaign, with the high-volume peritonitis group exceeding these levels, and these were also the groups with the highest mortality rates. Although our approach of two different basal rates of volume supply can be criticized, it should be noted that even animals in the high-volume groups received additional fluid boluses as a result of the appearance of clinical signs of hypovolemia. In clinical sepsis trials, the total amount of fluid given is rarely indicated. It is evident that high targets for filling pressures will result in large amounts of administered fluids when capillary leakage is present, and the administered fluid does not translate into a significant increase in venous return. For example, in the study by Rivers and colleagues [7], patients received a mean (± standard deviation) of 5 (± 3) liters of fluid within the first six hours. In other patient groups, including patients with multiorgan failure and sepsis, patients received 13 to 30 liters of fluid for resuscitation within 24 hours [22,23]. There is growing evidence that large amounts of fluids may be harmful, especially in septic patients [11,24,25], but also in other patient groups [22]. Our results point in the same direction.

Many of the experimental sepsis studies, including the present one, have used substantially larger doses of HES than is recommended in the clinical setting. Recent trials in clinical sepsis have found a dose-related association between HES and renal failure in sepsis [26]. Although a different HES solution was used in the present study, we cannot exclude that HES influenced the outcomes due to its pharmacological properties. Nevertheless, urinary output increased and creatinine concentrations decreased in both control and endotoxin high-volume groups. Furthermore, histology revealed major abnormalities in the endotoxin high-volume group but not in the peritonitis high-volume group.

Mitochondrial dysfunction has been suspected to contribute to mortality in sepsis. We found that neither the models of sepsis nor the volume resuscitation strategy resulted in altered hepatic or muscle mitochondrial complex I- and II-dependent respiration. We cannot exclude sepsis-induced impairment of mitochondrial function by mechanisms not tracked by our methods [27-29]. Nevertheless, normal arterial lactate concentrations and hepatic vein lactate/pyruvate ratios in all groups do not seem to suggest major mitochondrial respiration abnormality either. Recently, energetic failure of peripheral blood mononuclear cells in sepsis has been implicated in the modulation of immune response [30]. Nevertheless, how volume overload potentially aggravates early immune suppression remains unclear.

The relevance of our results for clinical sepsis deserves consideration. Although both sepsis models have many similarities with clinical sepsis, there are important differences, both in the models *per se *and in the treatments tested. First, both models included major abdominal surgery before induction of sepsis. The impact of recent surgery on metabolic demands and blood flow will inevitably be superimposed on the effects of sepsis. Second, the volume support was started at the same time that sepsis was induced, whereas clinical sepsis is typically associated with a delay in starting the treatment. Third, early antibiotics improve the outcome of clinical sepsis, but this was not included in our treatment. Fourth, hypotension not responsive to fluids alone is treated with vasoactive agents in clinical sepsis. As we did not use any inotropes or vasopressors, this clearly limits the extrapolation of our results to clinical sepsis.

## Conclusions

We conclude that aggressive volume resuscitation initially maintains systemic hemodynamics and regional blood flow in experimental endotoxemia and fecal peritonitis. However, it markedly increases mortality. Supplemental fluids should be used only as long as tissue perfusion can be improved. Future experiments should more closely mimic the natural course and treatment of sepsis.

## Key messages

• Aggressive volume resuscitation increases mortality in experimental sepsis.

• Mitochondrial complex I- or II-dependent muscle and hepatic respiration is maintained after 24 hours of endotoxemia and fecal peritonitis.

## Abbreviations

ANOVA: analysis of variance; HES: hydroxyethyl starch; H&E: hematoxylin and eosin.

## Competing interests

The authors declare that they have no competing interests.

## Authors' contributions

SMJ and JT designed the study, supervised the experiments, and revised the manuscript. SB, HB, FP, VK, JG, VK, and LBH conducted the experiments, including anesthesia. SB drafted the manuscript. TR performed the statistical analysis. TR, FP, SD, and EB performed the mitochondrial experiments. SD and UK performed the remaining laboratory analyses. LEB and GB performed surgery and revised the manuscript. PL supervised all laboratory analysis and revised the manuscript. LW performed all histological analyses. All authors read and approved the final manuscript.

## Supplementary Material

Additional file 1 A Word file containing a table that lists additional methods, along with related references. Click here for file

Additional file 2 A Word file containing four tables. Table S1 lists acid-base-balance and oxygen transport parameters. Table S2 gives hepatic mitochondrial ATP/ADP and ADP/oxygen ratios and calculated maximal ATP production obtained from mitochondrial respiration analysis. Table S3 lists skeletal muscle ATP content obtained from biopsies and muscle ATP/ADP ratios. Table S4 gives details of regional blood flows. Click here for file

Additional file 3 A PDF file containing six figures. Figure S1 is a comparison of complex I- and II-dependent hepatic mitochondrial respiration between the groups. Figure S2 shows lactate/pyruvate ratios in the hepatic vein. Figure S3 is a comparison of complex I- and II-dependent muscle mitochondrial respiration between the groups. Figure 4 shows lung histology: colloid plaques. Figure S5 shows lung histology: atelectasis. Figure S6 shows liver histology. Click here for file
