# Supplementary material for: Effect of fluid resuscitation on mortality and organ function in experimental sepsis models
Source: Crit Care. 2009 Nov 23;13(6):R186. doi: 10.1186/cc8179 (PMC2811934; doi:10.1186/cc8179)
Supplement: Additional file 1 — A Word file containing a table that lists additional methods, along with related references. [file cc8179-S1.rtf]

EFFECT OF FLUID RESUSCITATION ON MORTALITY AND ORGAN FUNCTION IN EXPERIMENTAL SEPSIS MODELS


Additional data file #1


Sebastian Brandt, Tomas Regueira, Hendrik Bracht, Francesca Porta, Siamak Djafarzadeh, 
Jukka Takala, José Gorrasi, Erika Borotto, Vladimir Krejci, Luzius Hiltebrand, 
Lukas E. Bruegger, Guido Beldi, Ludwig Wilkens, Philipp M. Lepper, Ulf Kessler, 
Stephan M. Jakob

Materials and methods
The animals were fasted for 12 hours prior to the experiment, with free access to water.
Animal preparation and experimental setting
Since prolonged septic state is a typical risk factor for acute lung injury, the minute ventilation, FiO2, and PEEP of the animals were adjusted following a standardized protocol (PEEP 5-14 cm H20) with the aim of keeping PaO2 levels between 13.3 kPa (100 mmHg) and 20 kPa (150 mmHg), PaCO2 levels between 4.5 and 5.5 kPa (34–41 mmHg), and the inspiratory plateau pressure below 35 cm H2O. When plateau pressure increased above 35 cm H2O, the tidal volume was reduced (minimally 6 ml/kg). When compliance decreased, a recruitment maneuver was performed, and when resistance increased, the animals were intra-tracheally suctioned. In addition, a standardized recruitment maneuver in pressure-controlled ventilation was performed after 6 hours of the stabilization period, and after 12 hours of the experimental protocol (peak pressures of 30 cm H2O for 10 seconds, and with 10 min intervals). 
The jugular veins were cannulated via a midline cervical incision for the placement of hepatic venous and pulmonary artery catheters, and another fluid-filled catheter was placed in the left carotid artery for blood pressure measurement and blood sampling. Afterward, a midline laparotomy was performed, and a urinary catheter was inserted into the bladder.
Fluid-filled catheters were inserted into the renal, mesenteric and portal veins and into the hepatic artery. Afterwards, ultrasound Doppler flow probes (Transonic® Systems Inc., Ithaca, NY, USA) which had been previously calibrated in vitro were positioned around the right carotid artery, the celiac trunk, the superior mesenteric, splenic and hepatic arteries, and the portal vein. Regional blood flows and hepatic and portal venous blood pressures were measured continuously during the experiment (WinDaq®, DATAQ instruments, Akron, OH, USA). Occluders were placed around superior mesenteric artery, portal vein and celiac trunk. The hepatic buffer response was tested at 0, 3, 6, 12, 18 and 24 hours. An occluder around the superior mesenteric artery was inflated and hepatic artery pressure was equalized with baseline by occluding the celiac trunk. Muscle and subcutaneous tissue PO2 were measured by intramuscularly (M. quadriceps femoris) inserted polarographic catheters (Licox® PO2 probe, GMS, Kiel, Germany) and recorded continuously (Biopac AcqKnowledge 3.7®, Goleta, CA, USA). Using jejunostomy and gastrostomy, customized fluid-filled microdialysis catheters—as described by Knuesel et al. [31]—and tonometers (Tonocap®, Datex-Ohmeda, Helsinki, Finland) were inserted into the jejunum and stomach. Laser Doppler needle and surface probes (Optronics®, Oxford, UK) were inserted into the liver and kidney, and fixed on the surface of gastric and jejunal mucosa and of the kidney. A fluid-filled catheter was inserted into the pericardial space through the diaphragm. This catheter was not used in this experiment, but was placed because these animals were also included in a second experiment in which other animals were randomized to cardiac tamponade. Hemodynamic (heart rate, carotid and pulmonary artery pressures and central venous pressure, continuous cardiac output) and respiratory (FiO2, airway pressures, tidal volume, mean airway pressure, end tidal CO2) parameters and fluid volume input and output were monitored and recorded in a clinical information management system (Clinisoft, GE Healthcare®, Helsinki, Finland). This system stores two-minute median values to minimize the number of artifacts. In addition, intermittent cardiac output (4°C bolus thermodilution technique; S/5 Compact Critical Care Monitor; Datex-Ohmeda®, Helsinki, Finland) and pulmonary artery occlusion pressure were measured hourly and also recorded in the clinical information management system. An esophageal Doppler probe (Deltex®, Chichester, UK) for measurement of aortic blood flow was inserted via the mouth and advanced into the esophagus until an optimal Doppler signal was observed. Central temperature, recorded from the thermistor of the pulmonary artery catheter (CCO/SvO2 catheter; Edwards Lifesciences®, Munich, Germany) and peripheral skin temperature (tip of a toe) were also continuously recorded in the clinical information management system. Finally, Ch 32 ascites drains were inserted via both flanks of the animals. After these procedures, the abdominal wall was tightly approximated with clamps and covered with wet towels.
Once the experiment was started, manipulation was avoided to minimize the possibility of biosensor displacement. At the end of the experiment, the correct position of each sensor was controlled visually.

Fluid challenge
After six hours of stabilization a fluid challenge was performed to study the effect of rapid infusion of 2 x 200 ml HES in the postoperative period. 

Skeletal muscle mitochondrial isolation
At baseline, at 6 hours, and at the end of the experiment, skeletal muscle tissue samples were taken from the quadriceps muscle in the living animal for isolation of mitochondria. Muscle specimens were rapidly immersed in ice-cold muscle isolation buffer (KCl 100 mmol/L, MgSO4 10 mmol/L, morpholinopropane sulfonic acid 50 mmol/L, Ethylene-glycol-tetra-acetate (EGTA) acid 1.0 mmol/L, and ATP 1.1 mmol/L, pH 7.4), transported to the laboratory, and weighed [32, 33]. After several rinses with isolation buffer, the skeletal muscle was minced using scissors and was suspended in 10 ml/g of tissue of the same medium and treated with a protease (Sigma-Aldrich, St. Louis, MO, USA), 5 mg/g mince for 10 min at 4°C, with constant stirring. Afterwards, the suspension was diluted with 10 ml/g of isolation medium supplemented with defatted bovine serum albumin (BSA) (0.2%), and homogenized in a Potter Elvehjem homogenizer with a loose-fitting Teflon pestle (ten strokes). The supernatant was separated by centrifugation (10 min at 10,000 g), and the pellet was resuspended in BSA-supplemented isolation medium (10 ml/g tissue). The suspension was centrifuged for 10 min at 2,500 g, the supernatant was filtered through two layers of gauze, and the mitochondria were sedimented at 7,700 g for 10 min. The mitochondria were subjected to two additional washes using 5 ml BSA-supplemented muscle isolation buffer/g tissue and 2.5 ml of 100 mmol/L KCl, 50 mmol/L morpholinopropane sulfonic acid, and 0.5 mmol/L EGTA, pH 7.4, buffer/g muscle, and finally resuspended in ~1.0 mL of 100 mmol/L KCl, 50 mmol/L morpholinopropane sulfonic acid and 0.5 mmol/L EGTA, pH 7.4. 

Hepatic mitochondrial isolation
At the end of the experiment, hepatic tissue samples were taken in the living animal for isolation of liver mitochondria. Isolation of liver mitochondria was performed at 4°C using a standard procedure based on differential centrifugation [33]. The samples of liver (15 g) excised at the end of the experiment were rapidly immersed in ice-cold liver isolation buffer (mannitol 220 mmol/l, sucrose 70 mmol/l, morpholinopropane sulfonic acid 5 mmol/l, pH 7.4), minced with scissors, and homogenized with an additional 10 ml/g of homogenization media (liver isolation buffer plus EGTA 2 mmol/l) in a Potter Elvehjem homogenizer with a loose-fitting Teflon pestle (four strokes). The homogenate was then centrifuged for 10 minutes at 700 g. The supernatant was collected and centrifuged again for 10 minutes at 7,000 g. The supernatant was discarded at this time; the pellet was then resuspended in isolation buffer and centrifuged twice for 10 minutes at 7,000 g for further purification of the mitochondria. The pellets were then suspended in buffer at a final concentration of 50–100 mg mitochondrial protein per milliliter.

Determination of mitochondrial respiration
Protein concentration for both muscle and liver was determined spectrophotometrically with the Biuret method using bovine serum albumin as a standard. Respiratory rates were determined at a final mitochondrial protein concentration of 1 mg/ml by using a Clark electrode (Yellow Springs Instruments) in an oxygraphic chamber (Yellow Springs Instruments, Yellow Springs, OH, USA) at 37ºC with continuous stirring. The medium used for respiration measurements consisted of KCL 25 mmol/l, morpholinopropane sulfonic acid 12.5 mmol/l, ethylene glycol-bis N,N,N',N' –tetraacetic acid 1 mmol/l and potassium phosphate buffer 5 mmol/l (pH 7.4). Rates of respiration are given in nanoatoms of O2 per minute per mg of mitochondrial protein. Maximal oxidative capacities were determined in the presence of saturating concentrations of O2, ADP (0.2 mM) and specific mitochondrial substrates. Substrates used were glutamate (20 mM), which provides nicotinamide adenine dinucleotide (NADH) to the respiratory chain (complex I-dependent respiration), and succinate (20 mM), which provides flavin adenine dinucleotide (FADH) to the respiratory chain (complex II-dependent respiration). The coupling of phosphorylation to oxidation was determined by calculating the respiratory control ratio [34] as the ratio between ADP-stimulated respiration (state 3) and respiration after ADP depletion (state 4). In addition, the added ADP/consumed oxygen ratio (ADP:O; nanomol/nanoatom) for glutamate- and succinate-dependent respiration was calculated, and the maximal ATP production (ADP:O ratio * state 3 respiration) was derived [35].

Blood sampling
Blood samples for the measurement of hemoglobin and blood gases were taken at baseline and at 3, 6, 12 and 24 hours from pulmonary and carotid arteries and from portal, hepatic and mesenteric veins (ABL 520 and OSM 3 [pig module]; Radiometer®, Copenhagen, Denmark, and YSI 2300 Stat Plus®, Yellow Springs Instruments, Yellow Springs, Ohio, USA). Creatinine, aspartate amino-transferase, alanine amino-transferase, creatinine kinase, and creatinine kinase - MB were measured from carotid arterial samples at baseline and at the end of the experiment.


Histological analysis
Immediately after the animals died, tissue specimens from the lung, kidney and liver were harvested and fixed with 4% formaldehyde solution for 24 hours. Subsequently, the samples were dehydrated and embedded in paraffin. After hematoxylin/eosin staining, the histological specimens were analyzed by an experienced pathologist (LW). First, the pathologist reviewed some of the specimens of the control moderate-volume group to evaluate potential effects of surgery and prolonged anesthesia. Afterwards, all specimens (including those of the control moderate-volume group) were randomized and analyzed blindly. For kidney specimen analysis, three categories were defined as follows: (1) no significant changes: tubular epithelial cells revealed slight changes in morphology only; (2) slight damage: presence of tubular epithelial cell vacuolization and necroses; (3) severe damage: majority of tubular epithelial cells with vacuolization and necrosis. For liver specimen analysis, histological abnormalities were classified in six categories: (1) prominence of portal tracts due to increased eosinophilic staining of hepatocytes in Rappaport zone I; (2) pericentral dilated sinusoids; (3) dilated sinusoids in all Rappaport zones; (4) pronounced pericentral hepatocyte vacuolization; (5) vacuolization of hepatocytes in all Rappaport zones; (6) pericentral necrosis. Lung specimens were classified according to presence or absence of atelectasis and of colloid plaques.

Causes of mortality
For the classification of mortality, four investigators, blinded to the experimental group, independently classified the mechanisms of death retrospectively based on graphical trends of physiological variables from the clinical information system and laboratory data. The classification was clinically oriented and included three options: acute cardiac failure (e.g., low cardiac output, acute right heart failure, arrhythmia), vasoplegia (prolonged systemic hypotension with maintained or increased cardiac output) and respiratory failure (prolonged severe arterial hypoxemia without primary hemodynamic compromise). In case of disagreement, the cases were re-reviewed, and the pathophysiology discussed among the investigators until agreement was obtained.
