# Supplementary material for: Effect of fluid resuscitation on mortality and organ function in experimental sepsis models
Source: Crit Care. 2009 Nov 23;13(6):R186. doi: 10.1186/cc8179 (PMC2811934; doi:10.1186/cc8179)
Supplement: Additional file 2 — A Word file containing four tables. Table S1 lists acid-base-balance and oxygen transport parameters. Table S2 gives hepatic mitochondrial ATP/ADP and ADP/oxygen ratios and calculated maximal ATP production obtained from mitochondrial respiration analysis. Table S3 lists skeletal muscle ATP content obtained from biopsies and muscle ATP/ADP ratios. Table S4 gives details of regional blood flows. [file cc8179-S2.rtf]

EFFECT OF FLUID RESUSCITATION ON MORTALITY AND ORGAN FUNCTION IN EXPERIMENTAL SEPSIS MODELS


Additional data file #2


Sebastian Brandt, Tomas Regueira, Hendrik Bracht, Francesca Porta, Siamak Djafarzadeh, 
Jukka Takala, José Gorrasi, Erika Borotto, Vladimir Krejci, Luzius Hiltebrand, 
Lukas E. Bruegger, Guido Beldi, Ludwig Wilkens, Philipp M. Lepper, Ulf Kessler, 
Stephan M. Jakob


Table S1:
Acid-base-balance and oxygen transport parameters.
Values are mean ± SD.

Variable	Group	N	Baseline	3 hours	6 hours	12 hours	End	Interactions	p	
Arterial pH								Time x model effect:
	p=0.005
	
	C 10 ml/kg	8	7.46 ± 0.02	7.46 ± 0.03	7.44 ± 0.04	7.47 ± 0.04	7.42 ± 0.08			
	C 20 ml/kg	8	7.46 ± 0.02	7.46 ± 0.02	7.47 ± 0.02	7.45 ± 0.10	7.41 ± 0.07			
	E 10 ml/kg	7	7.49 ± 0.02	7.45 ± 0.03	7.47 ± 0.04	7.47 ± 0.05	7.44 ± 0.10			
	E 20 ml/kg	7	7.48 ± 0.02	7.44 ± 0.04	7.46 ± 0.03	7.43 ± 0.04	7.44 ± 0.06			
	P 10 ml/kg	7	7.47 ± 0.03	7.38 ± 0.05	7.39 ± 0.05	7.42 ± 0.02	7.34 ± 0.07	ANOVArm P:	p=0.001	
	P 20 ml/kg	8	7.48 ± 0.02	7.41 ± 0.06	7.43 ± 0.04	7.45 ± 0.05	7.31 ± 0.09			
Base excess								Time x model effect:
	p=0.005
	
(mmol·L-1)	C 10 ml/kg	8	4 ± 1	4 ± 1	3 ± 3	5 ± 2	4 ± 3			
	C 20 ml/kg	8	3 ± 2	3 ± 2	3 ± 3	3 ± 3	2 ± 6			
	E 10 ml/kg	7	5 ± 1	2 ± 1	4 ± 1	4 ± 2	4 ± 3			
	E 20 ml/kg	8	4 ± 2	3 ± 2	3 ± 2	4 ± 1	4 ± 3			
	P 10 ml/kg	8	4 ± 1	0 ± 2	0 ± 2	1 ± 1	0 ± 4	ANOVArm P: 	p=0.001	
	P 20 ml/kg	8	4 ± 1	3 ± 2	3 ± 2	4 ± 2	1 ± 3			
Systemic DO2
(ml·kg-1·min-1)								Time x model effect:
	p=0.001
	
	C 10 ml/kg	8	11.1 ± 2.4	10.6 ± 2.5	11.6 ± 3.1	12.6 ± 5.1	11.6 ± 2.1			
	C 20 ml/kg	8	9.4 ± 3.1	10.0 ± 1.9	10.7 ± 2.5	11.2 ± 3.1	11.7 ± 3.3			
	E 10 ml/kg	7	9.4 ± 1.9	9.9 ± 3.5	11.5 ± 3.2	12.7 ± 2.5	14.0 ± 3.6	ANOVArm E:	p=0.002	
	E 20 ml/kg	8	10.7 ± 2.4	10.5 ± 3.4	13.0 ± 4.5	16.3 ± 4.5	13.3 ± 2.9			
	P 10 ml/kg	8	10.6 ± 2.0	15.0 ± 4.6	15.6 ± 2.9	11.9 ± 4.2	12.0 ± 3.3	ANOVArm P: 	p=0.001	
	P 20 ml/kg	8	9.5 ± 1.9	15.4 ± 4.6	14.2 ± 3.3	11.8 ± 1.4	13.4 ± 6.1			
Systemic VO2
(ml·kg-1·min-1)								Time effect:
	p=0.02
	
	C 10 ml/kg	8	4.80 ± 0.51	4.43 ± 0.86	4.75 ± 0.84	5.50 ± 2.71	4.66 ± 0.83			
	C 20 ml/kg	8	4.45 ± 1.15	3.96 ± 0.56	4.24 ± 0.90	4.20 ± 0.70	4.76 ± 1.31			
	E 10 ml/kg	7	4.76 ± 1.03	4.73 ± 1.56	4.93 ± 1.01	5.12 ± 1.10	5.77 ± 0.98			
	E 20 ml/kg	7	5.29 ± 1.34	4.98 ± 1.25	4.94 ± 1.35	5.11 ± 1.30	5.82 ± 1.87			
	P 10 ml/kg	8	4.80 ± 1.13	5.52 ± 0.94	5.37 ± 1.95	4.90 ± 1.20	5.52 ± 0.98			
	P 20 ml/kg	8	4.70 ± 0.43	5.54 ± 1.00 	5.49 ± 1.10	4.75 ± 1.30	5.76 ± 1.33			

C: controls; E: endotoxin; P: peritonitis

Table S2: 
Hepatic mitochondrial ATP/ADP (median (range)) and ADP/O ratios and calculated maximal ATP production obtained from mitochondrial respiration analysis.
Values are mean ± SD.

	Control 10 ml/kg	Control 20 ml/kg	Endotoxin 10 ml/kg	Endotoxin 20 ml/kg	Peritonitis10 ml/kg	Peritonitis 20 ml/kg	p	
ATP/ADP ratio	0.39 (0.15-0.74)	0.29 (0.20-0.45)	0.35 (0.09-1.51)	0.27 (0.10-0.31)	0.36 (0.06-1.50)	0.47 (0.30-1.18)	0.3	
ADP/O ratio								
Complex I	2.3 ± 0.5	2.2 ± 0.5	2.3 ± 0.6	2.3 ± 0.6	2.3 ± 0.8	3.0 ± 0.6	M= 0.14
V= 0.3
MxV= 0.2	
Complex II	1.7 ± 0.2	1.4 ± 0.4	1.6 ± 0.3	1.7 ± 0.8	1.8 ± 0.4	1.9 ± 0.4	M= 0.4
V= 0.8
MxV= 0.4	
Maximal ATP production								
Complex I	152 ± 56	145 ± 45	148 ± 104	102 ± 30	123 ± 58	237 ± 116	M= 0.16
V= 0.37
MxV= 0.02*	
Complex II	251 ± 137	185 ± 62	201 ± 101	113 ± 48	212 ± 109	244 ± 101	M= 0.15
V= 0.2
MxV= 0.3	

ATP and ADP: Adenosine triphosphate and diphosphate, respectively.
ADP/O ratio expressed in nanomol/nanoatom. ATP production expressed in nanomol*nanoatom/min/mg.
Statistics: For ATP/ADP ratio p-values correspond to Kruskal-Wallis test between the groups. For ADP/O ratio and maximal ATP production p-values correspond to univariate analysis of variance. M: Model, V: Volume, MxV: model x volume interaction.
*: Post hoc test, p=0.043 between peritonitis moderate vs. high volume.


Table S3. 
Skeletal muscle ATP content and muscle ATP/ADP ratios. 
Values are mean ± SD.

Variable	Group	Baseline	End	p value*	
ATP content 
(µmol/g wet tissue)				time = 0.1
time x model = 0.07
time x volume = 0.04 (a)
time x volume x model = 0.09	
	Control 10 ml/kg	5.4 ± 1.1	5.9 ± 1.2		
	Control 20 ml/kg	5.4 ± 1.2	5.2 ± 1.3		
	Endotoxin 10 ml/kg	5.6 ± 1.5	4.4 ± 2.6		
	Endotoxin 20 ml/kg	4.3 ± 1.3	5.1 ± 1.7		
	Peritonitis10 ml/kg	5.9 ± 1.4	2.8 ± 2.6		
	Peritonitis 20 ml/kg	5.9 ± 1.5	5.6 ± 0.9		
ATP/ADP ratio				time = 0.9
time x model = 0.4
time x volume = 0.24
time x volume x model = 0.033 (b)	
	Control 10 ml/kg	6.4 ± 0.8	7.3 ± 1.4		
	Control 20 ml/kg	6.6 ± 0.9	6.0 ± 0.7		
	Endotoxin 10 ml/kg	6.7 ± 1.1	7.2 ± 3.8		
	Endotoxin 20 ml/kg	6.4 ± 1.2	7.3 ± 1.0		
	Peritonitis10 ml/kg	6.6 ± 1.3	3.7 ± 2.8		
	Peritonitis 20 ml/kg	6.7 ± 1.5	8.1 ± 3.6		

ATP and ADP: Adenosine triphosphate and diphosphate, respectively
*refers to the repeated measures analysis of variance.
(a) Post-hoc test, p=0.051 between moderate and high-volume animals.
(b) Post-hoc test, p=0.037 between peritonitis moderate vs. high volume.


Table S4:
	
Regional blood flows. 
Values are mean ± SD.

Variable	Group	N	Baseline	3 hours	6 hours	12 hours	End	Interactions	p	
Renal artery flow
(ml·kg-1·min-1)								Time x model effect:
Time x volume effect:	p=0.001
p=0.046	
	C 10 ml/kg	8	5.2 ± 1.7	5.5 ± 1.8	5.4 ± 2.2	5.7 ± 2.4	5.8 ± 2.8			
	C 20 ml/kg	8	4.9 ± 1.4	5.6 ± 1.7	6.6  ± 2.3	6.7 ± 1.7	6.6 ± 2.7			
	E 10 ml/kg	7	4.5 ± 1.8	4.3 ± 1.2	5.9 ± 2.7	6.9 ± 3.0	5.8 ± 2.1	ANOVArm E: 	p=0.015	
	E 20 ml/kg	8	4.8 ± 3.2	5.0 ± 3.3	6.3 ± 4.6	6.1 ± 4.0	3.6 ± 2.3			
	P 10 ml/kg	8	5.5 ± 1.6	4.7 ± 1.2	4.0 ± 1.0	4.0 ± 1.8	3.0 ± 1.6	ANOVArm P:	p=0.024	
	P 20 ml/kg	8	5.6 ± 2.3	5.8 ± 2.1	6.2 ± 2.7	5.2 ± 2.3	2.8 ± 1.7	ANOVArm high-volume:	p=0.006	
Hepatic artery flow (ml·kg-1·min-1)								Time x model effect:
	p=0.003
	
	C 10 ml/kg	8	3.3 ± 1.5	4.1 ± 2.5	4.1 ± 2.2	4.4 ± 2.3	4.5 ± 1.2	ANOVArm C: 	p=0.03	
	C 20 ml/kg	8	3.8 ± 1.4	4.4 ± 1.4	4.9 ± 1.9	5.4 ± 2.0	5.2 ± 2.8			
	E 10 ml/kg	7	2.9 ± 0.8	2.1 ± 1.0	3.1 ± 1.4	5.9 ± 2.8	6.6 ± 2.2	ANOVArm E: 	p=0.006	
	E 20 ml/kg	8	4.1 ± 1.7	3.4 ± 2.4	3.3 ± 2.2	6.5 ± 4.5	6.4 ± 4.8			
	P 10 ml/kg	8	4.3 ± 3.3	4.3 ± 3.0	4.2 ± 3.1	5.5 ± 3.9	3.8 ± 2.3			
	P 20 ml/kg	8	3.6 ± 2.0	3.7 ± 1.8	4.3 ± 1.8	4.0 ± 2.2	3.7 ± 2.6			
C: controls; E: endotoxin; P: peritonitis
