# Supplementary material for: Effect of fluid resuscitation on mortality and organ function in experimental sepsis models
Source: Crit Care. 2009 Nov 23;13(6):R186. doi: 10.1186/cc8179 (PMC2811934; doi:10.1186/cc8179)

EFFECT OF FLUID RESUSCITATION ON MORTALITY AND ORGAN FUNCTION IN EXPERIMENTAL SEPSIS MODELS

Additional data file #3

Sebastian Brandt, Tomas Regueira, Hendrik Bracht, Francesca Porta, Siamak Djafarzadeh, Jukka Takala, José Gorrasi, Erika Borotto, Vladimir Krejci, Luzius Hiltebrand, Lukas E. Bruegger, Guido Beldi, Ludwig Wilkens, Philipp M. Lepper, Ulf Kessler, Stephan M. Jakob

Figure captions

Figure S1:

Comparison of complex I- and II-dependent hepatic mitochondrial respiration between the groups.

Top: Maximal hepatic mitochondrial complex I- and II-dependent respiration (state 3)

Middle: Resting hepatic mitochondrial complex I- and II-dependent respiration (state 4)

Bottom: Respiratory control ratio (RCR) for complex- I and II-dependent respiration.

Black (□) and open (o) circles represent non-survivors and survivors, respectively.

Statistics refers to: Univariate analysis of variance. Model refers to the effect of the model of sepsis. Volume refers to the effect of volume strategy. Model x Volume refers to the interaction of the model and volume.

For complex I state 4 model effect p=0.046, post hoc test (independent t-test): p=0.009 between endotoxin and control groups (*). Non-survivors had a lower complex II-dependent RCR (p=0.01, independent t-test).

Figure S2:

Lactate/Pyruvate ratio (hepatic vein).

Statistics refers to: Repeated measures ANOVA. *Time x Model* refers to the effect of the model of sepsis in time. *Time x Volume* refers to the effect of volume strategy in time.

Time x Model x Volume refers to the interaction of the model and volume in time.

Figure S3:

Comparison of complex I- and II-dependent muscle mitochondrial respiration between the groups.

Top: Complex I-dependent maximal (state 3) and resting (state 4) muscle mitochondria respiration

Bottom: Complex II-dependent maximal (state 3) and resting (state 4) muscle mitochondria respiration

Black (□) and open (o) circles represent non-survivors and survivors, respectively.

Repeated measures ANOVA was used for the statistics. Time x Model refers to the effect of the model of sepsis in time. Time x Volume refers to the effect of volume strategy in time.

Time x Model x Volume refers to the interaction of the model and volume in time.

For complex I state 3 time x volume effect p=0.04, post hoc test (repeated measures ANOVA): p=0.024 between moderate and high-volume groups. Non-survivors had a tendency towards lower complex I- and II-dependent maximal respiration (state 3) (p=0.09 and p=0.07, respectively).

Figure S4:

Lung histology: colloid plaques (%).

Figure S5:

Lung histology: atelectasis (%).

Figure S6:

Liver histology, comparison between groups.

A: Septation

B: Pericentral dilatation of sinusoids

C: Generalized dilatation of sinusoids

D: Pericentral hepatocytes vacuolisation

E: Generalized hepatocytes vacuolization

F: Pericentral necrosis

Figure S1: Hepatic mitochondrial respiration

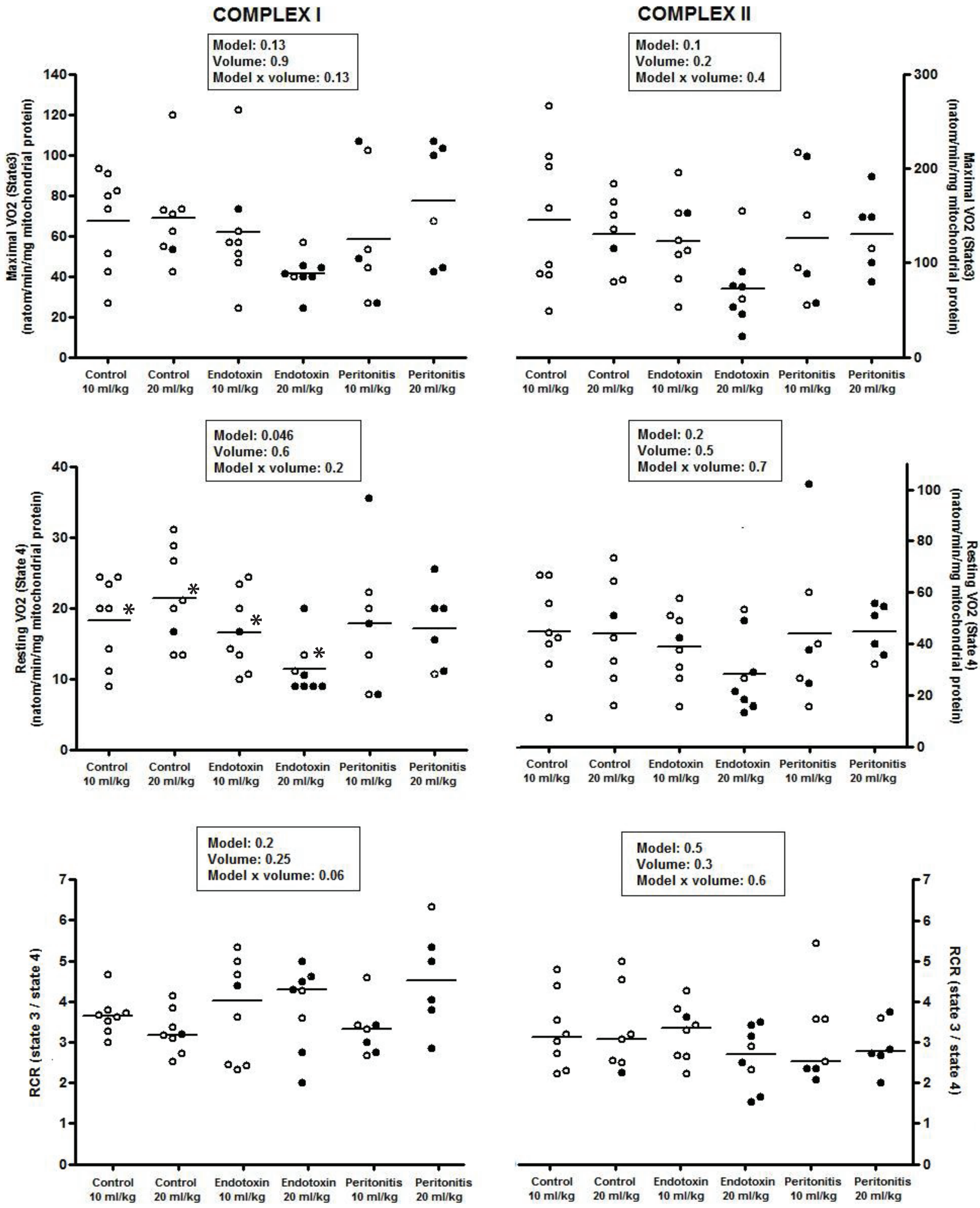

Figure S2: Lactate / Pyruvate ratio (hepatic vein)

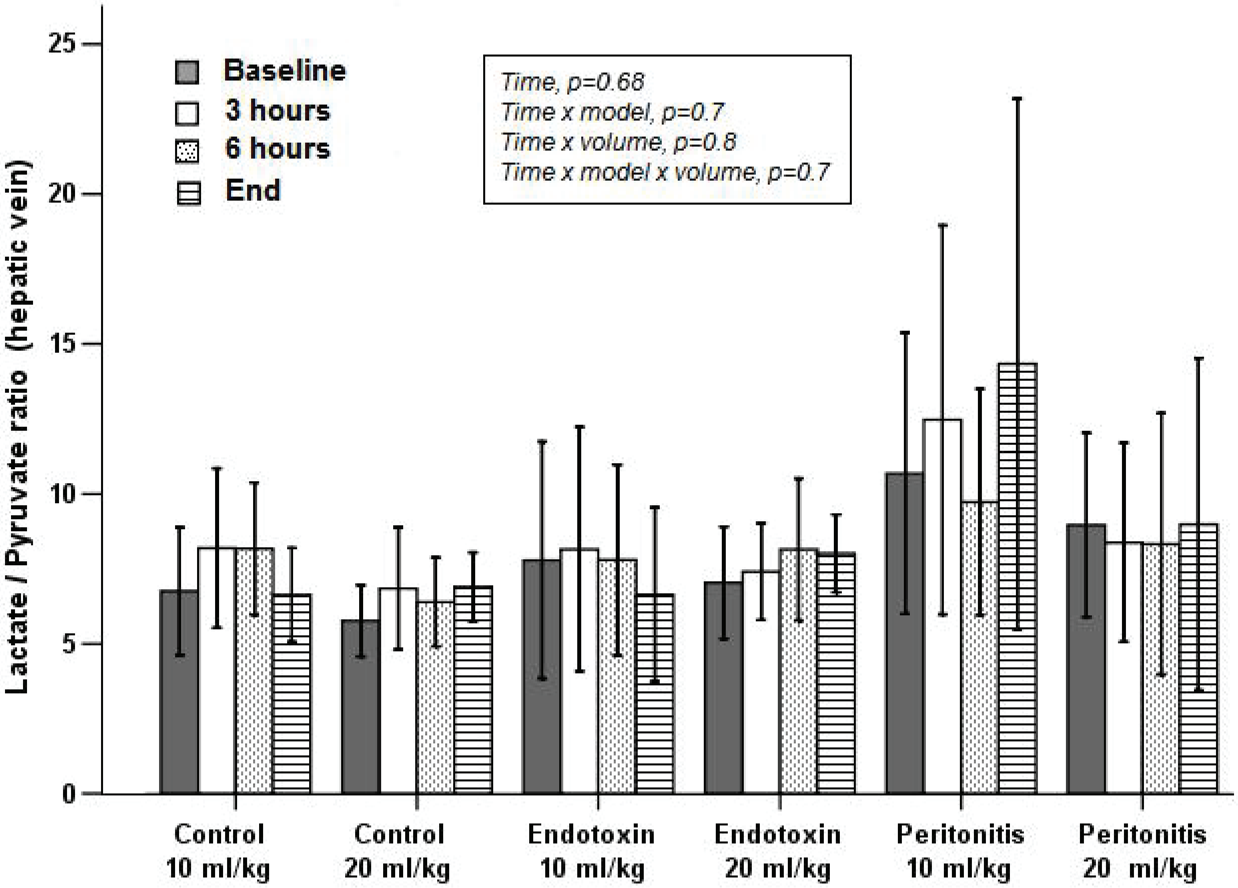

Figure S3: Muscle mitochondrial respiration

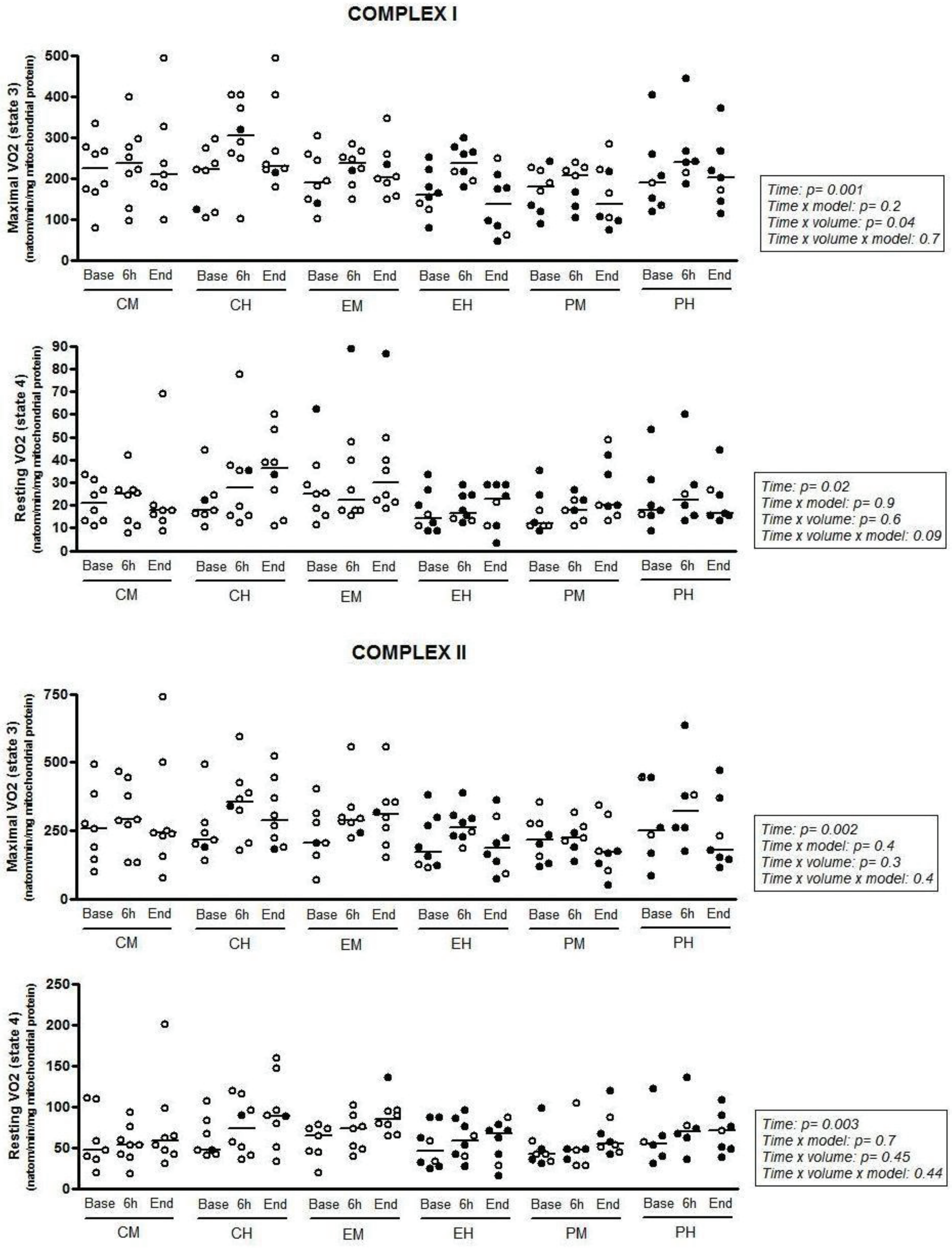

Figure S4 Percentage of colloid plaques (lung)

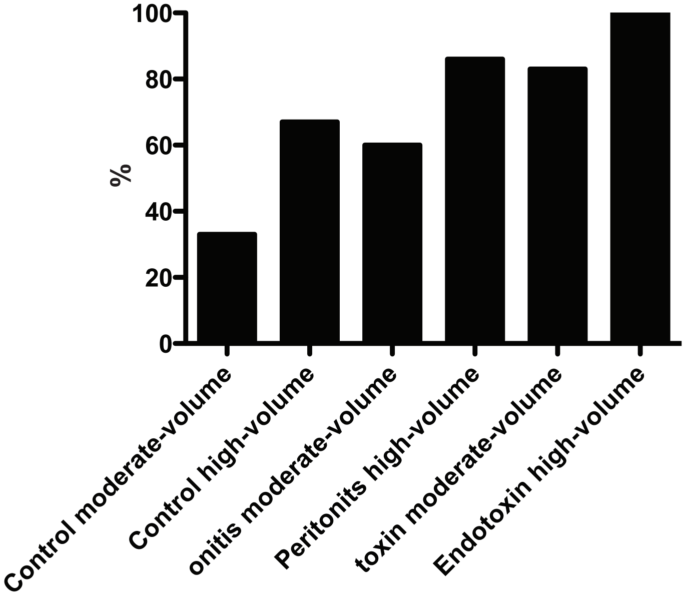

Figure S5 Percentage of atelectasis

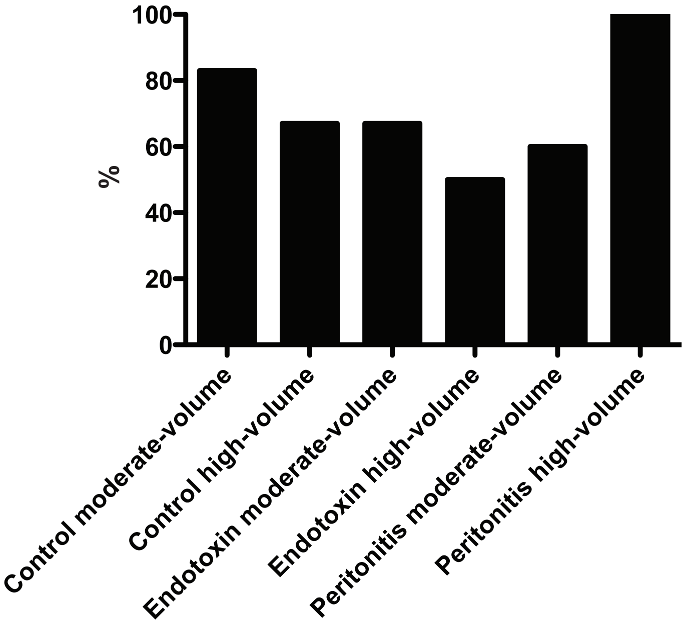

Figure S6 Liver histology

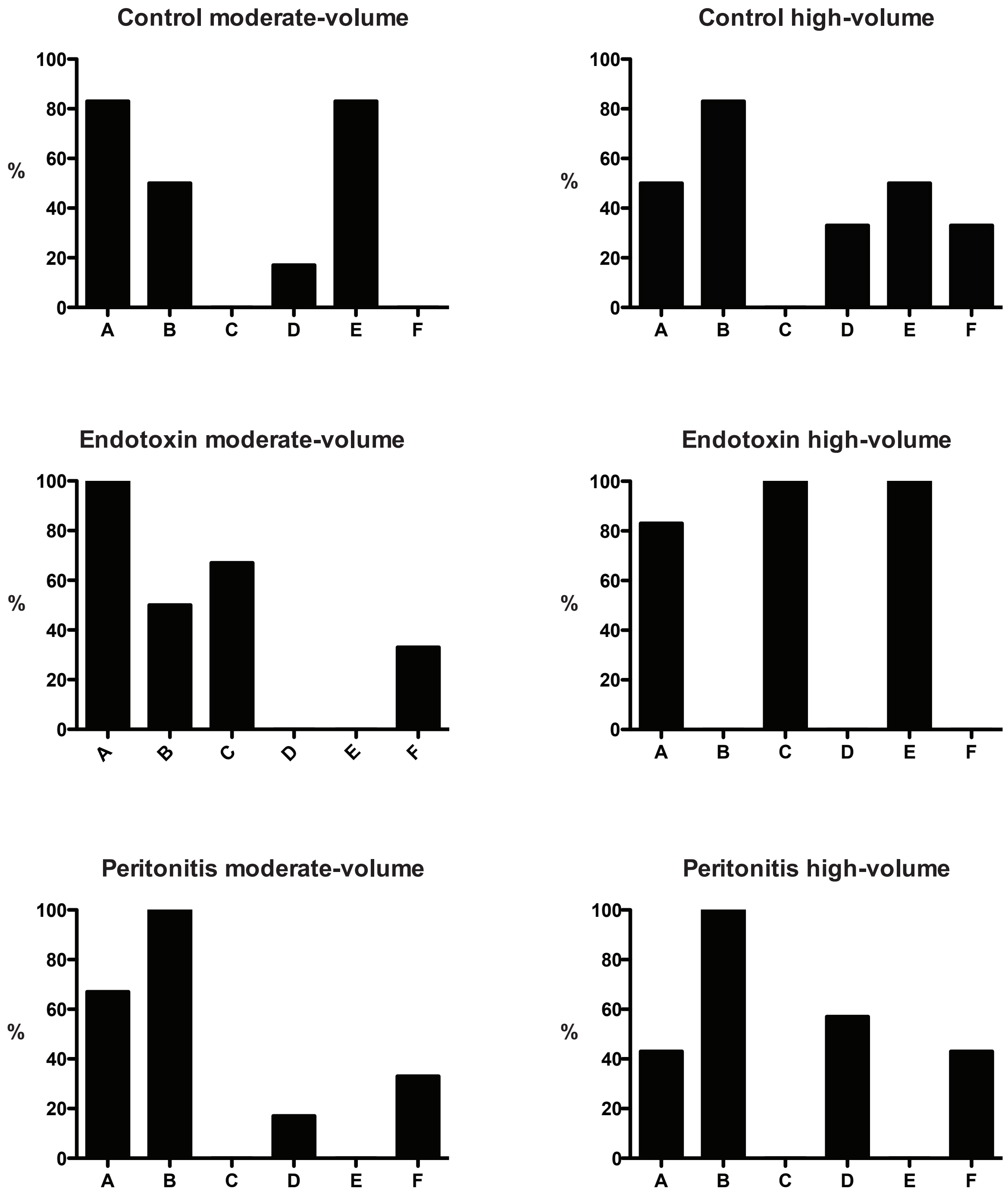

Supplement: Additional file 3 — A PDF file containing six figures. Figure S1 is a comparison of complex I- and II-dependent hepatic mitochondrial respiration between the groups. Figure S2 shows lactate/pyruvate ratios in the hepatic vein. Figure S3 is a comparison of complex I- and II-dependent muscle mitochondrial respiration between the groups. Figure 4 shows lung histology: colloid plaques. Figure S5 shows lung histology: atelectasis. Figure S6 shows liver histology. [file cc8179-S3.pdf]
